# Percolation-theoretic bounds on the cache size of nodes in mobile opportunistic networks

**DOI:** 10.1038/s41598-017-05988-5

**Published:** 2017-07-18

**Authors:** Peiyan Yuan, Honghai Wu, Xiaoyan Zhao, Zhengnan Dong

**Affiliations:** 1 Henan Normal University, School of Computer and Information Engineering, Xinxiang, 453000 China; 2 Henan University of Science and Technology, Information Engineering College, Luoyang, 471000 China; 3 National Digital Switching System Engineering and Technological Research Center, 450000 Zhengzhou, China

## Abstract

The node buffer size has a large influence on the performance of Mobile Opportunistic Networks (MONs). This is mainly because each node should temporarily cache packets to deal with the intermittently connected links. In this paper, we study fundamental bounds on node buffer size below which the network system can not achieve the expected performance such as the transmission delay and packet delivery ratio. Given the condition that each link has the same probability *p* to be active in the next time slot when the link is inactive and *q* to be inactive when the link is active, there exists a critical value *p*_*c*_ from a percolation perspective. If *p* > *p*_*c*_, the network is in the supercritical case, where we found that there is an achievable upper bound on the buffer size of nodes, independent of the inactive probability *q*. When *p* < *p*_*c*_, the network is in the subcritical case, and there exists a closed-form solution for buffer occupation, which is independent of the size of the network.

## Introduction

Current portable/wearable devices have been integrated with many sensors and wireless functions, such as WiFi and Bluetooth, which confers on them powerful capabilities, especially in sensing, computing and communication. The more powerful these devices become, the more likely it is that they share contents locally, leading to the emergence of mobile opportunistic networks (MONs)^1^. MONs use node mobility to provide occasional contact opportunities for mobile devices to transmit packets. Such a new networking paradigm employs device-to-device communication, which is rarely considered in traditional infrastructure-based networks, to alleviate the overloading criticality and enhance the coverage areas. On the other hand, in infrastructure-less areas, MONs may be the only communication solution. These benefits enable numerous applications, including rural communications^2^, wild monitoring^3^, crowd sensing^4^ and metropolitan awareness of issues^5^.

Compared to traditional wireless networks, one of the features of MONs is that the intermittent connectivity is mainly caused by broken links due to some external factors varying in different scenarios. For example, in an ad hoc mobile vehicle network^6^, two vehicles are connected when they enter the communication range of each other, and the link between them is disconnected when they move outside the transmission range. In^7^, S. Wang, J. Zhao and L. Tong studied a cognitive radio network where the activity of primary users is driven by an ON-OFF process such that the secondary users should keep inactive until the primary users switch from the ON to OFF mode.

The factors mentioned above are external in some sense, which implies that link disconnection and inactivity can not be eliminated by improving internal conditions such as enhancing the network capacity or node process speed. With external constraints, node buffer size can not approach zero even if the network capacity approaches infinity, since each node should temporarily cache packets to deal with the broken links. Therefore, there exists a performance limit on node buffer occupation. On the other hand, current research communities mainly focus on the data forwarding schemes of MONs (e.g., PROPHET^8^, Bubble^9^, Spray-Wait^10^, SMART^11^ and Hotent^12^). Some analytical results with respect to flooding time^13^, network diameter^14^, and delay-capacity tradeoff^15^ have also been obtained, providing valuable insights into data forwarding efficiency and system performance. However, such insights may only reflect one aspect of MONs without considering the influence of node buffer size.

In this paper, we study node buffer occupation in MONs, where broken links are caused by node mobility or a channel access scheme. We assume that the system capacity can be regarded as infinite, compared to the low packet generation rate. By using this assumption, we try to illustrate that even if the system capacity is unlimited, there still exists a lower bound on node buffer size below which the network system cannot achieve the suggested performance, such as the transmission delay and packet delivery ratio. We use the edge-Markovian dynamic graph to model MONs. This model can reproduce the power law + exponential tail distribution of the pairwise node inter-contact time, which has been observed in several real data traces of MONs^16^. Starting from an arbitrary initial probability distribution of links, at every time slot, links change their states (active or inactive) based on a two-state Markovian process with probabilities *p* (active) and *q* (inactive). If a link is inactive at slot *t*, it will be active at slot *t* + 1 with probability *p*. If, instead, the link is active at slot *t*, it switches to the inactive state at the next slot with probability *q*. From the perspective of percolation theory, there is a critical value *p*_*c*_ for *p*. If *p* > *p*_*c*_, the network is in the supercritical case, and there exists a connected giant cluster, a unique infinite connected cluster at any time, with a high probability when the number of nodes becomes infinity. In contrast, when *p* < *p*_*c*_, the network is in the subcritical case, and the giant cluster disappears almost certainly when the number of nodes goes to infinity. Fundamental limits on node buffer size are quite different in the two cases. In the supercritical case, there is an upper bound on the buffer size of nodes, independent of the inactive probability *q*, while in the subcritical case, there exists a closed-form solution for buffer occupation that is independent of the size of the network.

The rest of this paper are organized as follows. We first review related works and introduce the preliminaries. Then, we analyze the node buffer occupation in the supercritical case and present the results in the subcritical case. After that, we conduct the experiment and analyze the results, followed by a short conclusion.

## Related Works

MONs is a natural evolution from traditional mobile ad hoc networks. In MONs, the links are intermittently connected due to node mobility and power on/off, mobile nodes communicate with each other opportunistically and route packets in a store-carry-and-forward style. In the past several years, much effort has been expended to improve the performance of opportunistic routing algorithms in terms of reducing the forwarding delay or increasing the packet delivery ratio. Some valuable results have been achieved that provide theoretical guidance for performance optimization. We introduce them in detail.

### Cache-aware Opportunistic Routing Algorithms

Considering the limited buffer size of portable devices, cache-aware solutions become very important in MONs. A. Balasubramanian *et al*. take MONs routing as a resource allocation problem and turn the forwarding metric into per-packet utilities that incorporate two factors: one is the expected contribution of a packet if it were replicated, and the other is the packet size. The utility, then, is the ratio of the former factor over the latter, which determines how a packet should be replicated in the system^17^. To deal with the short contacts and fragmented bundles, M. J. Pitkanen and J. Ott^18^ integrated application level erasure coding on top of existing protocols. They used Reed Solomon codes to divide single bundles into multiple blocks and observed that the block redundancy increased the catch hit rate and reduced the response latency.

S. Kaveevivitchai and H. Esaki proposed a message deletion strategy for a multi-copy routing scheme^19^. They employed extra nodes deployed at the system’s hot regions to relay the acknowledgement (ACK) messages, and copies matching the ID of ACK messages are dropped from the buffer. A. T. Prodhan *et al*. proposed TBR^20^, which ranks messages with their TTL, hop count and number of copies. A node will delete the copy of a message if it receives a higher priority message and its buffer is full. Recently, D. Pan *et al*.^21^ developed a comprehensive cache schedule algorithm that integrates different aspects of storage managements including queue strategy, buffer replacement and redundancy deletion.

### Performance Analysis of Cache-aware Opportunistic Routing Algorithms

Some analytical results mainly focus on metrics such as the flooding time^13, 22, 23^, network diameter^14^ and delay-capacity tradeoff^15^, in which the buffer size of nodes is usually assumed to be unlimited. Several works discuss the congestion issue with the epidemic algorithm. For example, A. Krifa *et al*.^24^ proposed an efficient buffer management policy by modeling the relationship between the number of copies and the mean delivery delay/rate. When a new packet copy arrives at a node and the node finds its buffer full, it drops the packets with the minimal marginal utility value. G. Zhang and Y. Liu employed revenue management and dynamic programming to study the congestion management strategy of MONs^25^. Given a class of utility functions, they showed that one arrived packet should be accepted and that the solution is optimal if and only if the value of the benefit function is greater than that of the cost function.

The authors of^26^ evaluated the impact of buffer size on the efficiency of four kinds of routing algorithms. They observed that these protocols reacted differently to the increase of buffer size in mobile vehicle networks. Generally speaking, both the Epidemic and MaxProp benefit from the increased buffer size on all nodes (i.e., the mobile and terminal nodes). PROPHET and SprayWait instead have no significant improvement when only the buffer size of terminal nodes increases. X. Zhuo *et al*.^27^ explored the influence of contact duration on data forwarding performance. To maximize the delivery rate, they modeled the data replication problem with mixed integer programming technology, subject to the storage constraint.

## Preliminaries

### External Factors and Broken Links

In this paper, we study the general influence of external factors on the network connectivity rather than focusing on a specific type of external constraint. At each time slot, each link switches between the inactive state and active state. Two endpoints of a link can transmit or receive packets only if the link is active.

**Figure 1:**
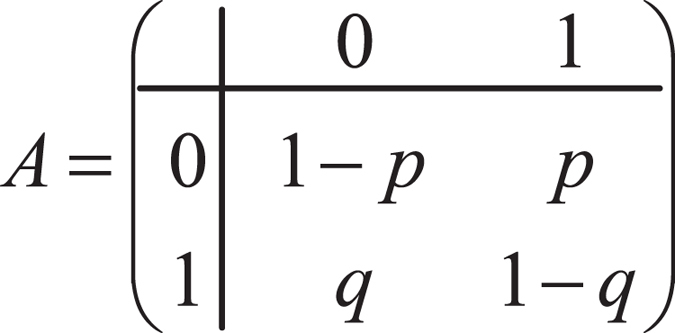
Transition matrix.

We model the external factors in the network with the edge-Markovian dynamic graph (EMDG), which implies that: (1) States of each link vary from one time slot to another, and are i.i.d. among time slot *t* + 1 and the time slots before *t*. (2) The probabilities of being active or inactive are two constants *p* and *q* for all links, respectively. Mathematically, let *n* denote the number of nodes and 0 ≤ *p*, *q* ≤ 1. The EMDG can be denoted by ${\mathscr{G}}(n,p,q,L(t))$ if

$$L(t)=\{l\in (\begin{array}{l}n\\ 2\end{array})\,:{X}_{t}(l)=1\}$$

where $$\{{X}_{t}(l)\,:l\in (\begin{array}{l}n\\ 2\end{array})\}$$ are independent Markov chains with the transition matrix and *l* is the edge of EMDG. As shown in Fig. 1, the transition matrix indicates the transition of the link state in the next time slot, where 0 and 1 denote that the link is inactive or active, respectively. For example, the element *a*_12_ means the link is inactive and will become active in the next time slot with probability *p*.

### Percolation of Connected Links

Considering the possibility of link activity, we can not guarantee a connected path for each source-destination pair at all times. However, since the states of links are i.i.d, there exists a critical probability ${p}_{c}=c\frac{\mathrm{log}\,n}{n}$ such that (please refer to property 3.5 of ref. 28):

#### Supercritical case

If $p\ge c\frac{\mathrm{log}\,n}{n}(c > \mathrm{1)}$, then w.h.p. ${\mathscr{G}}(n,p,q,L(t))$ is in the supercritical case and there exists a connected giant cluster.

#### Subcritical case

There is a constant *c* ≤ 1/2 such that for $p\le c\frac{\mathrm{log}\,n}{n}$, ${\mathscr{G}}(n,p,q,L(t))$ is in the subcritical case and the giant cluster disappears.

### Bounds on Node Buffer Size in the Supercritical Case

In this section, we study the bounds on buffer size of nodes in ${\mathscr{G}}(n,p,q,L(t))$ when $p\ge c\frac{\mathrm{log}\,n}{n}$. The main result is that the expected buffer size of nodes can be bounded within a certain range as stated in Theorem 1.

#### **Theorem 1**

*For a randomly selected node u of* ${\mathscr{G}}(n,p,q,L(t))$ if $p\ge c\frac{\mathrm{log}\,n}{n}$, at the end of a time slo*t t*,

1 $$E({B}_{u}(t))\ge r{T}_{d}\frac{p+q}{q}$$

*And in some forwarding schemes, it is*

2 $$E({B}_{u}(t))\le {c}_{1}r{T}_{d}\frac{\mathrm{log}\,n}{pM}$$

where the *E*(*B*_*u*_(*t*)) is a random variable that denotes the expectation of the node buffer size, *r* is the packet generation rate per node, *T*_*d*_ is the duration of one time slot, *c*_1_ is a constant and *M* is the number of nodes in the giant cluster.

### Proof of Inequality (1)

Here, we take the node *u* as an example. Suppose it belongs to the giant cluster, it does not need to buffer packets when the links connecting it are active. This is mainly because there exists a connected path with a high probability between the node *u* and other nodes; the transmission delay hence tends to zero. Instead, it at least needs to store packets generated by itself when links are inactive. The expected waiting time before links become active is 1/*π*_1_, where *π*_1_ belongs to the stationary distribution *π*, which is a special distribution for a Markov chain such that if the chain starts with its stationary distribution, the marginal distribution of all states at any time will always be the stationary distribution. The variables *i* and *j* denote the rows and columns in the transition matrix above, so as to compute the transition probability of the link stage in the next time slot. *π*_*j*_ refers to *π*_1_ and *π*_2_, which denotes the value of the stationary distribution of a Markov chain. With the transition matrix *A*, we have:

$$\{\begin{array}{l}{\pi }_{j}=\sum _{i=1}^{2}{\pi }_{i}{a}_{ij},j=1,2\\ {\pi }_{j} > 0,\sum _{j=1}^{2}{\pi }_{j}=1\end{array}$$

where *a*_*ij*_ denotes elements of *A*. We obtain ${\pi }_{1}=\frac{q}{p+q}$. Applying Little’s Law,

$$E({B}_{u}(t))\ge r{T}_{d}\frac{p+q}{q}$$

According to Little’s Law, the long-term average number of customers in a stable system is equal to the long-term average effective arrival rate multiplied by the average time a customer spends in the system. Note that $\frac{p+q}{q}$ is the expected number of time slots before links become active, ${T}_{d}\frac{p+q}{q}$ is the average time a customer spends in the system, and *r* is the arrival rate. Hence, we obtain the number of packets generated by *u* with equation (1).

In the following subsections, we first design an optimal forwarding scheme, and then prove that with this scheme, the buffer occupation specified in equation (2) can be achieved.

### Optimal Forwarding Scheme (OFS)

**Figure 2:**
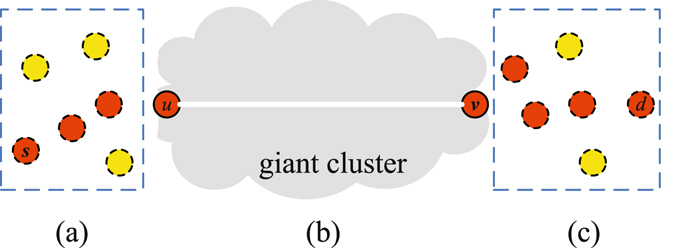
Three stages in the optimal forwarding scheme.

Nodes in the supercritical case can be classified into two types based on whether they belong to the giant cluster. One type is connected nodes, which are in the giant cluster, and the other type is disconnected nodes. The former constitutes a connected component and the packets can be transmitted fast. In contrast, the disconnected nodes need larger buffer sizes than the connected nodes since they have to store packets before the links turn active. The worst case is that both the source *s* and the destination *d* are disconnected nodes and are just separated by the giant cluster, as shown in Fig. 2. There exist three stages in the optimal forwarding scheme: the source flooding stage, the shortest path stage and the destination flooding stage. We first introduce the source flooding stage.

**Figure 3:**
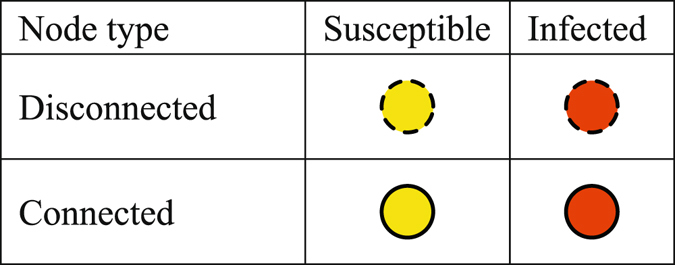
Four types of nodes.

1. The source flooding stage. In general, the flooding scheme achieves the highest packet delivery ratio and the lowest transmission delay, but it requires the largest storage space, which is necessary for our study, since we focus on the fundamental bounds on node buffer size especially in the worst case. Considering that source *s* and its neighbors are disconnected nodes, it needs to flood packets generated by itself and received from its neighbors. In the flooding scheme, if there is a packet at node *u* at time slot *t* − 1, node *u* will send the packet to all the nodes that are connected to *u* at time slot *t* if the link between them is active. To characterize the flooding process, we use an infectious disease-diffusion algorithm^29^ and further classify nodes into a susceptible state and an infected state. We call a node infected, if it carries a packet and the node is susceptible if it does not carry the packet. Then, there exist four kinds of nodes: disconnected and susceptible (*DS*), disconnected and infected (*DI*), connected and susceptible (*CS*), and connected and infected (*CI*), as shown in Fig. 3.

With the disease-diffusion algorithm, node *s* first sends copies of a packet *m* to its neighbors. The newly infected neighbors then repeat the same process until any node that belongs to the giant cluster receives *m*, as shown in Fig. 2(a). At the end of this stage, the infected nodes (i.e., several disconnected nodes and one connected node) constitute the source expanding tree, *SET*.

(2) The shortest path stage. Since there exists a connected path for each source-destination pair with a high probability in the giant cluster, we use the shortest path to transmit packets in this stage. Suppose a node *u* in the *SET* belongs to the giant cluster. It sends *m* to node *v* along the shortest path as shown in Fig. 2(b). In other words, if there is a packet at time slot *t* − 1 at node *u*, at time slot *t* node *u* instantaneously sends the packet to the next hop in the shortest path if the link is active.
(3) The destination flooding stage. After the node *v* receives the message *m*, it diffuses *m* among the disconnected nodes until *m* reaches the destination node *d*. Finally, nodes having a copy of *m* form the destination expanding tree, *DET*.

With OFS, it can be intuitively inferred that the buffer occupation of disconnected nodes is limited. This is mainly because in each time slot, the giant cluster exists, and the size of *SET* and *DET* are finite. Therefore, the disconnected nodes only need to buffer messages coming from nearby sources or going to nearby destinations.

### Contact Probability between disconnected and connected nodes

Let *C*(*n*, *p*, *q*, *L*(*t*)) denote the set of connected nodes and *D*(*n*, *p*, *q*, *L*(*t*)) the set of disconnected nodes (i.e., a node is a connected node if it belongs to the giant component; otherwise, it is a disconnected node). Let *w*_1_ ∈ *D*(*n*, *p*, *q*, *L*(*t*)), *w*_2_ ∈ *C*(*n*, *p*, *q*, *L*(*t*)) and ${l}_{{w}_{1}{w}_{2}}\in L$. For any *t*_0_, *t* (*t* > *t*_0_), define the set of nodes in D that have been connected to C in at least one time step *i* ∈ {*t*_0_ + 1, …, *t*_0_ + *t*}

$${H}_{{t}_{0}t}=\{{w}_{1}\in D\backslash C\,:{X}_{i}({l}_{{w}_{1}{w}_{2}})=1,{w}_{2}\in C\}$$

where ${l}_{{w}_{1}{w}_{2}}(i)=1$ denotes that the link between *w*_1_ and *w*_2_ is active at moment *i*.

The following lemma is a key component in proving equation (2). Roughly speaking, it states that the probability that one disconnected node does not have contact with one connected node during [0, *t*] (i.e., the link between them remains inactive within time step *t*) decreases exponentially in *t*.

#### **Lemma 1**

*Let w*_1_ ∈ *D*\*C, for any t*_0_ ≥ 0 and *t* ≥ 1. *It holds that*

3 $$P({w}_{1}\notin {H}_{{t}_{0}t})\le {e}^{-ptM}$$

where *M* is the size of *C*.

#### **Proof:**

For any node *w*_2_ ∈ *C*, the event ${w}_{1}\notin {H}_{{t}_{0}t}$ indicates that there is no active link between *w*_1_ and *w*_2_ during [0, *t*]. From the EMDG, the link inactivity probability is (1 − *p*), and we obtain ${{\rm{P}}({\rm{l}}}_{{{\rm{w}}}_{{\rm{1}}}{{\rm{w}}}_{{\rm{2}}}}{({\rm{i}})=0|{\rm{l}}}_{{{\rm{w}}}_{{\rm{1}}}{{\rm{w}}}_{{\rm{2}}}}({\rm{i}}-1)=0)={\rm{1}}-{\rm{p}}$.

Therefore,

$$\begin{array}{rcl}P({w}_{1}\notin {H}_{{t}_{0}t}) & = & P(\mathop{\cap }\limits_{{w}_{2}\in C}\underset{i={t}_{0}+1}{\overset{{t}_{0}+t}{\cap }}{X}_{i}({l}_{{w}_{1}{w}_{2}})=0)\\  & = & P(\mathop{\cap }\limits_{{w}_{2}\in C}{(1-p)}^{t})\end{array}$$

Since Markov chains of different links are independent, it holds that

$$P({w}_{1}\notin {H}_{{t}_{0}t})={(1-p)}^{tM}\le {e}^{-ptM}$$

### Finite Transmission Time

#### **Lemma 2**

*Let t*_*s → u*_ *denote the transmission time from node s to node u, which represents the time when the package is first transmitted from the source node into nodes belonging to the giant cluster. It satisfies*

4 $$P({t}_{s\to u}\ge \frac{\mathrm{log}\,n}{pM})\le \frac{1}{n}$$

#### **Proof:**

Recall that in stage 1 of the OFS, the flood process will stop if one *DI* node encounters any *CS* node. Assume there are *k DI* nodes and all of them will encounter the *CS* node *u*. The elapsed times are *t*_1,*u*_, …, *t*_*k*,*u*_. Hence, ${t}_{s\to u}=\,{\rm{\min }}({t}_{\mathrm{1,}u},\ldots ,{t}_{k,u})$. On the other hand, from lemma 1, we know that there exists one node *j* ∈ *D*(*n*, *p*, *q*, *L*(*t*)). It will contact the node *u* after $\frac{\mathrm{log}\,n}{pM}$ steps with at least probability 1 − O(1/*n*). This is mainly because

$$P(j\in {H}_{{t}_{0}t})\ge 1-{e}^{-ptM}\ge 1-{e}^{-\mathrm{log}n}=1-\mathrm{1/}n$$

when $$t\ge \frac{\mathrm{log}\,n}{pM}$$.

Since *t*_*s* → *u*_ is the minimum of *k* elapsed time, ${t}_{s,u}\le \frac{\mathrm{log}\,n}{pM}$. We then obtain:

$$P({t}_{s,u}\ge \frac{\mathrm{log}\,n}{pM})=1-P({t}_{s,u}\le \frac{\mathrm{log}\,n}{pM})\le \frac{1}{n}$$

### Proof of Inequality (2)

Let *E*(*B*_*s*_(*t*)) denote the expectation of buffer occupation in stage 1. We have:

$$E({B}_{s}(t))\le r{T}_{d}{S}_{n}\frac{\mathrm{log}\,n}{pM}$$

For the buffer occupation in stage 3, we correspondingly have

$$E({B}_{d}(t))\le r{T}_{d}{D}_{n}\frac{\mathrm{log}\,n}{pM}$$

where *S*_*n*_ is the size of SET and *D*_*n*_ represents that of DET. Finally, the buffer occupation of node *u* with the OFS is the sum of the above two parts, $E({B}_{u}(t))\le r{T}_{d}({S}_{n}+{D}_{n})\frac{\mathrm{log}\,n}{pM}={c}_{1}r{T}_{d}\frac{\mathrm{log}\,n}{pM}$, where *c*_1_ is determined by *S*_*n*_ and *D*_*n*_. We will discuss the value of *S*_*n*_ and *D*_*n*_ in the evaluation section.

### Bound on Node Buffer Size in the Subcritical Case

If $p\le c\frac{\mathrm{log}\,n}{n}(c\le \mathrm{1/2)}$, the network is in the subcritical case, and the giant cluster does not exist. The main result is that there is a closed form for buffer occupation of nodes as shown in Theorem 2.

#### **Theorem 2**

*For a randomly selected node u of* ${\mathscr{G}}(n,p,q,L(t))$ *if* $p\le c\frac{\mathrm{log}\,n}{n}(c\le \mathrm{1/2)}$, *at the end of a time slot t*,

5 $$E({B}_{u}(t))=\frac{r{T}_{d}}{2p}$$

Similar to stages (1) and (3) of the OFS, we still use the infectious disease-diffusion algorithm to distribute copies, but this time the shortest path stage disappears. The flooding scheme therefore dominates the forwarding process.

### The Number of Average Copies of a Packet When It Is Delivered

Let *S*(*t*) denote the number of susceptible nodes at a moment *t* and *I*(*t*) denote the number of infected nodes. From the infectious disease-diffusion process, we obtain the varying rate of *I*(*t*):

6 $$I^{\prime} (t)=(n-\mathrm{1)}pSIp$$

#### **Proof:**

**Figure 4:**
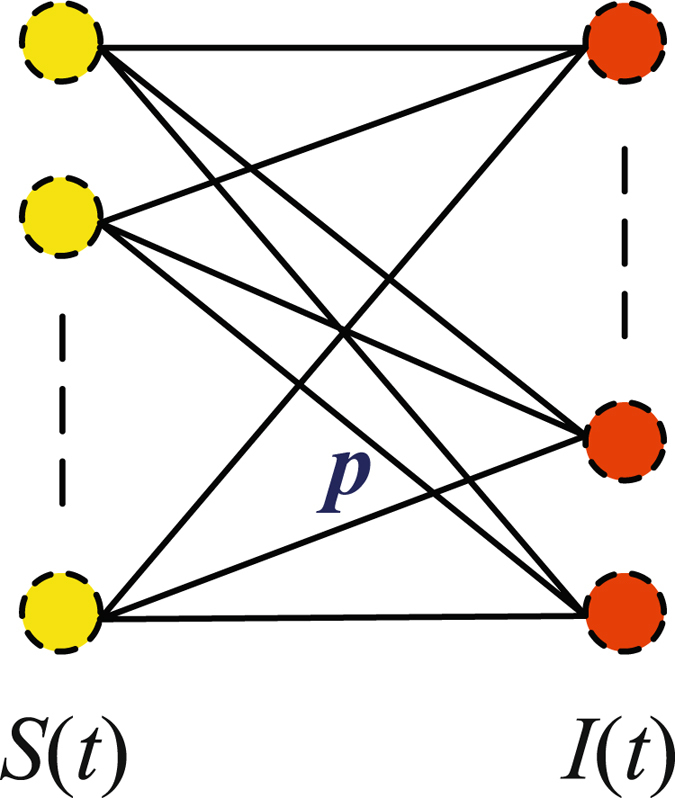
Number of active links.

Recall that with EMDG, the active probability of links is *p*, which implies that a node can contact (*n* − 1)*p* other nodes in one time slot. Since there are *I*(*t*) infected nodes and *S*(*t*) susceptible nodes, the number of active links among themis *S*(*t*) × *I*(*t*) × *p* as shown in Fig. 4. Hence, the number of new infected nodes in one time slot is (*n* − 1)*pSIp*.

With the condition *I*(0) = 1 and *I*(*t*) + *S*(*t*) = *n*, we obtain:

7 $$I(t)=\frac{n}{1+(n-\mathrm{1)}{e}^{-n(n-\mathrm{1)}t{p}^{2}}}$$

Correspondingly, the cumulative delay distribution ${\mathscr{P}}(t)$ until the first copy of a packet is delivered to its destination can be formulated as ${\mathscr{P}}^{\prime} (t)=(n-\mathrm{1)}pIp\mathrm{(1}-{\mathscr{P}})$. After some algebras, we obtain:

8 $${\mathscr{P}}(t)=1-(\frac{n}{(n-\mathrm{1)}+{e}^{n(n-\mathrm{1)}t{p}^{2}}})$$

The average number of copies of a packet when it is delivered to the destination, *E*(*c*) is hence derived:

9 $$E(c)={\int }_{0}^{\infty }I(t){\mathscr{P}}^{\prime} (t)dt-\mathrm{1=(}n-\mathrm{1)/2}$$

### Proof of equation (5)

We now prove equation (5).

Assume that each node is the source of one flow and the destination for another flow; then, there are *n* flows in the network. Given the (*n* − 1)/2 copies of a packet and the packet generation rate *r*, the number of packets generated by nodes is *nrT*_*d*_ and the total number of packets (the packets generated by nodes and their copies) in the system is *nrT*_*d*_(*n* − 1)/2, which is equally shared among the *n* nodes. The arrival rate of relay packets for each node is therefore (*n* − 1)*rT*_*d*_/2. Since a copy is deleted if the node encounters the destination, the contact rate is just the service rate (*n* − 1)*p*. According to the queuing theory, the average buffer size of nodes is the ratio of the arrival rate and service rate. Hence, we obtain $E({B}_{u}(t))=\frac{r{T}_{d}}{2p}$.

### Compute *S*_*n*_, *D*_*n*_ and *M*

We demonstrate how to compute the values of *S*_*n*_, *D*_*n*_ and *M* in this section.

Recall that *S*_*n*_ and *D*_*n*_ denote the size of SET and DET, respectively. Nodes in SET and DET are disconnected and do ont belong to the giant cluster *M*. Hence, the message diffusion in SET/DET also takes flooding in the same way as that in the subcritical case, and the number of infected nodes in SET/DET can be calculated by equation (7), where the parameters *n* and *p* are set in advance and the only issue is how to ascertain the value of *t*. Fortunately, from lemma 2, we know that the transmission time *t*_*s* → *u*_ can not exceed $\frac{\mathrm{log}\,n}{pM}$ with at least probability 1 − O(1/*n*). This means that the maximum value of *t* is $\frac{\mathrm{log}\,n}{pM}$, and we can use it to calculate the bound of the buffer size in equation (7). Using a similar process, we can get the value of *D*_*n*_.

For the number of connected nodes in the giant cluster (i.e., *M*), it can be obtained by the MATLAB function largestcomponent().

## Numerical Results

In this section, we present the simulation results to show the expectation of the buffer size in different conditions and other key components such as the number of nodes in the largest connected cluster.

For the supercritical case, we initially deploy 1000 nodes in the network. The value of *c* is 1.1 and *r* = *T*_*d*_ = 1. After that, we vary the parameter of *p* from 0.003 to 0.004 with a step 0.0005 to evaluate the network connectivity performance under different active probabilities. In addition, the simulation results are the average over 100 runs for statistical confidence.

**Figure 5:**
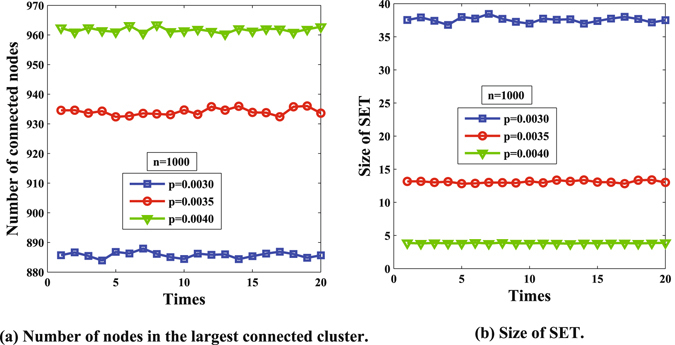
Number of nodes in the largest connected cluster and SET.

Figure 5(a) illustrates the number of connected nodes. It is obvious that the number of connected nodes ranges from 880 to 970 as the active probability increases from 0.0030 to 0.0040, which means there indeed exists a giant connected cluster in the network. The two parameters are positively related. That is, the smaller the value of *p* is, the smaller the value of *M* should be.

Figure 5(b) shows the size of SET. We note that in the supercritical case, the source flooding stage is very short and the number of nodes in SET is smaller compared to those in the giant cluster. For example, the size of SET is smaller than 40 in *p* = 0.003, and under the same condition, *M* tends to 890.

**Figure 6:**
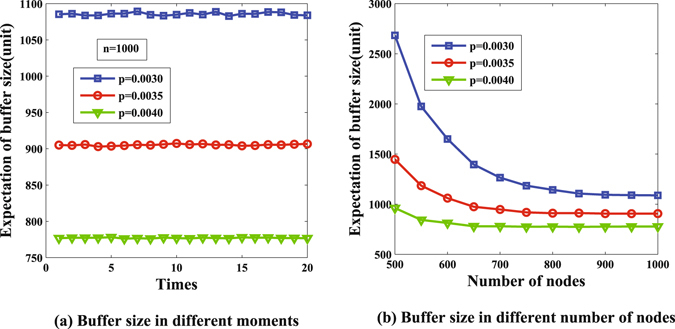
Buffer size in supercritical case.

After calculating the values of *S*_*n*_, *D*_*n*_ and *M*, we can compute the expectation of node buffer size by equation (2). The results are shown in Fig. 6(a,b). Figure 6(a) shows the expected buffer size at different moments, and there is a slight fluctuation. Figure 6(b) further shows the influence of the number of nodes on the expectation of buffer size. We observe that the expectation curve is declining sharply when the number of nodes increases from 500 to 700 and levels out at the end, which demonstrates that only bits of buffer size are needed in this scenario.

**Figure 7:**
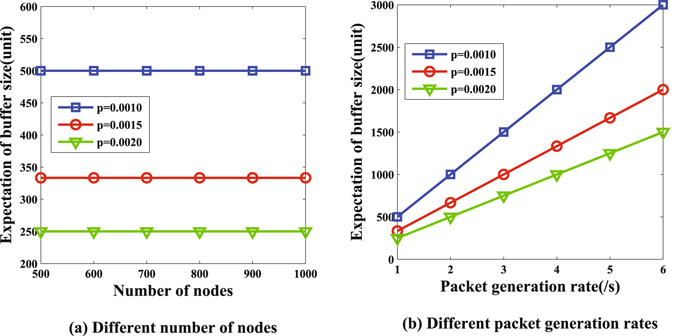
Buffer size in subcritical case.

For the subcritical case, we calculate the expectation of the node buffer size according to equation (5) and plot the results in Fig. 7, where the value of *p* is 0.001, 0.0015 and 0.002, respectively, and all of them satisfy the subcritical condition. Figure 7(a) shows the influence of *p* on buffer size, and Fig. 7(b) shows the role of packet generation *r*. The value of the buffer size ranges from 250 units to 500 with different active probability *p* and shows no relationship with the number of nodes in Fig. 7(a). In Fig. 7(b), the value of buffer size has a positive correlation with the parameter *r*. Generally speaking, the node buffer size is independent of the number of nodes in the subcritical case, which instead relies on the active probability *p*.

## Conclusion

In this paper, we study the cache potentiality of mobile opportunistic networks. We model the MONs with EMDG and find that there exists a critical probability *p*_*c*_ from the perspective of percolation theory. If the active probability *p* ≥ *p*_*c*_, the network is in the supercritical case and there exist two bounds on the buffer size of nodes: the lower bound is dependent on both the active probability *p* and the inactive probability *q*, while the upper bound is independent of *q*. If, instead, *p* ≤ *p*_*c*_, there is a closed-form solution for buffer occupation that, is independent of the size of the network. In the future, extensive simulations under various scenarios will be run to evaluate the results, and to explore the influence of different buffer management metrics.
